# Rheumatic Swellings Following Antitoxin Injections

**Published:** 1902-12

**Authors:** J. A. Metcalfe

**Affiliations:** Azusa, Cal.


### Rheumatic Swellings Following Antitoxin Injections.

AZUSA, CAL., September 2, 1902.

*Texas Medical Journal:*

Having spent a number of years in your dear old State, and feeling interested in everything pertaining to the welfare of its people, I gladly give you from my California home my experience with rheumatic swellings following antitoxin injections. Four years ago I had my first experience. After using four injections of 3000 units each, at intervals of eight hours, in a very severe case of laryngeal diphtheria membrane was cast off patient was doing finely, when in two and a half days she called for me. saying she was suffering very much. I found her wrists and ankles considerably swollen. This conditions increased and spread to all the joints of the limbs. Used salicylates. Patient was relieved entirely in one week. This patient was 20 years of age.

Second patient, January, 1902. Patient 10 years of age; severe laryngeal type. Used three injections antitoxin twelve hours apart, 3000 extra potent, then in twelve hours more 2000 units single x. Membrane all gone in twenty hours. Two days later membrance reformed on both tonsils; used two injections each 2000 units single x. Membrane gone entirely in twenty-four hours. When membrane was finally removed, there was partial paralysis of muscles of deglutition. Patient very weak and stomach in bad condition (very irritable). Three days after membrane was cleard away and five days after last injection patient had very severe joint swelling, but owing to the irritability of the stomach could not give the salicylates, but wrapped joints in hot flannels and cotton batting. In a week all swelling was gone and no soreness to speak of.

These cases were very interesting to me as I had used antitoxin in one hundred and fifteen cases (including seventy immunizations) with no such results in any other cases.

Will be glad to see any article you may have on this subject.

J. A. METCALFE, M. D.

